# Microwave-Assisted Catalytic Synthesis of Bio-Based Copolymers from Waste Cooking Oil

**DOI:** 10.3390/ma10030315

**Published:** 2017-03-20

**Authors:** Mahrzadi Noureen Shahi, Muhammad Arshad, Aman Ullah

**Affiliations:** 1 Department of Agricultural, Food and Nutritional Sciences, University of Alberta, Edmonton, AB T6G 2P5, Canada; mahrzadi@ualberta.ca (M.N.S.); arshad4@ualberta.ca (M.A.); 2 Department of Chemistry, The Islamia University of Bahawalpur, Bahawalpur 63100, Pakistan

**Keywords:** waste cooking oil, phthalic anhydride, copolymerization, microwaves

## Abstract

Solvent-free copolymerization of epoxides derived from fatty esters of waste cooking oil with phthalic anhydride using (salen)Cr^III^Cl as catalyst and n-Bu_4_NCl/DMAP (tetrabutylammonium chloride/4-(dimethylamino)pyridine) as co-catalysts was carried out for the first time under microwave irradiation, where reaction time was reduced from a number of hours to minutes. The polyesters were obtained with molecular weight (*M*w = 3100–6750 g/mol) and dispersity values (D = 1.18–1.92) when (salen)Cr^III^Cl/n-Bu_4_NCl was used as catalysts. Moreover, in the case of DMAP as a co-catalyst, polyesters with improved molecular weight (*M*w = 5500–6950 g/mol) and narrow dispersity values (D = 1.07–1.28) were obtained even at reduced concentrations of (salen)Cr^III^Cl and DMAP. The obtained products were characterized and evaluated by attenuated total reflection-Fourier transform infrared spectroscopy (ATR-FTIR), proton nuclear magnetic resonance (^1^H-NMR) spectroscopy, gel permeation chromatography (GPC), thermogravimetric analysis (TGA) and differential scanning calorimetric (DSC) Techniques.

## 1. Introduction

In recent years, utilization of renewable resources for the production of chemicals and polymeric materials have attracted the attention of researchers around the world because of their ease of availability, low cost and biodegradability and because they are an environmentally friendly alternative to overcome the deficiency of petroleum reservoirs [1,2,3,4,5,6]. Among different polymeric materials, polyesters from renewable resources have been explored extensively [7,8,9].

Recently, there have been various approaches and attempts described for the conversion of fatty acids from natural oils [10,11,12,13]. Fatty acid triglycerides from natural oils contain functional moieties, which are limited to certain types of reactions, and need chemical modification to improve their reactivity for the production of higher value products. Among these, epoxidation of fatty acids [14,15,16,17] plays an important role because of its highly reactive nature with most of the other substrates. The synthesis of polyesters from methyl epoxy stearate monomer should be promising, since the catalytic ring-opening copolymerization of epoxides with cyclic anhydrides, such as phthalic, maleic, or succinic anhydride, using different metal-complex systems is known and has been investigated in recent years [18]. A comparable effect on the use of microwave and conventional heating for ring opening polymerization of *L*-lactide by titanium complex has also been reported, where both of the methods provided a good molecular weight polylactide [19]. Alternating copolymerization of various epoxides and cyclic anhydrides with (salen)CrCl/onium salt as catalysts also yield polyesters with high molecular weights and narrow distribution values [20]. Among metal complexes, the chromium salen complex has proved to be the most effective catalyst for copolymerization of epoxides such as cyclohexene oxide, propylene oxide, styrene oxide, limonene oxide and anhydrides (succinic, maleic and phthalic anhydrides) [20,21,22].

As mentioned above, numerous examples have been reported for the utilization of natural oil-based monomers as starting materials. Methyl epoxystearate and other epoxidized fatty compounds have been used for the synthesis of a variety of polymers [16,23]; however, very little attention has been paid to utilize waste cooking oil as a renewable feedstock [24,25,26,27]. Waste cooking oil is mostly employed to produce biodiesel [28,29], while there is no example of using waste cooking oil into renewable polyesters. In the USA, around 100 million gallons per day of waste cooking oil is produced, as has been reported by the Energy Information Administration (EIA) department of the United States [30]. These large amounts of waste cooking oils are illegally dumped into rivers and landfills causing environmental pollution [31]. The use of waste cooking oil as a carbon source for the synthesis of polyesters turns the polluting domestic and restaurant waste into a valuable biodegradable product. The conversion of waste cooking oil into valuable biopolymers will eliminate the environmental impacts caused by the harmful methods of its disposal into drains.

Recently Biermann et al., reported the alternating ring-opening copolymerization of methyl 9,10-epoxystearate with various cyclic anhydrides to afford polyesters with longer reaction times (19–20 h) using conventional heating at high temperatures [14]. To overcome longer reaction times used in conventional heating, elevated temperature and higher catalyst ratio, for the first time, we are reporting ring opening copolymerization of epoxides derived from fatty esters of waste cooking oil with phthalic anhydride using (salen)Cr^III^Cl as catalyst and n-Bu_4_NCl/DMAP as co-catalyst under microwave irradiation in solvent-free conditions.

## 2. Experimental

### 2.1. Materials

Waste cooking oil was collected from a local restaurant in Edmonton, n-hexane, ethyl acetate, potassium hydroxide (KOH), dichloromethane (DCM, ≥99.5%), sodium chloride, anhydrous sodium saulphate, 3-chloroperbenzoic acid (≥99%), 4-(dimethylamino)pyridine (DMAP, ≥99%), silica gel for column chromatography (60 Å, 70–230 mesh, Whatman, Maidstone, UK) were purchased from Sigma-Aldrich (Oakville, ON, Canada) and were used as received. (1R,2R)-(−)-[1,2-Cyclohexanediamine-*N*,*N*’-bis(3,5-di-t-butylsalicylidene)]chromium(III) chloride [(salen)Cr^III^Cl] was purchased from Strem Chemicals (Newburyport, MA, Canada). Methanol (≥99.8%), tetrabutylammonium chloride (95%) and tetrahydrofuran (THF) were purchased from Fisher Scientific (Burlington, ON, Canada).

### 2.2. Methods

All reactions were performed in 10 mL sealed tube on CEM-Discover (120 V, Matthews, NC, USA), a source of microwave irradiation. While infrared mode was used to measure the temperature of the reaction contents.

Thin layer chromatography (TLC, Merck, Darmstadt, Germany) was performed on silica gel 60 F_254_ Aluminium plates. ATR-FTIR measurements were conducted on Bruker Alpha FTIR spectrophotometer (Bruker Optics, Esslingen, Germany) equipped with a single-bounce diamond ATR crystal. All Spectra were recorded at 16 scans and resolution of 4 cm^−1^ over the frequency range of 400–4000 cm^−1^. Spectra were acquired using OPUS software version 6.5 provided by Bruker. Spectral examination, measurements, and processing were carried out using Nicolet Omnic software. Gel permeation chromatography of the polymers was carried out on the system equipped with styragel HR1 GPC column and detector (ELSD2000s, Santa Clara, CA, USA). The injected volume of sample was 10 µL with 0.5 mg·mL^−1^. THF was used as eluent at a flow rate of 0.5 mL/min. ^1^H-NMR spectra were recorded in deuterated chloroform on Varian Inova (400 MHz) instrument (Palo Alto, CA, USA). Differential scanning calorimetric (DSC) analysis of selected copolymers were conducted on calorimetric apparatus (2920 Modulated DSC, TA Instrument, New Castle, DE, USA) using a stream of nitrogen gas. As all derived copolymers have low transition temperatures, so the samples were run within a temperature range of −40–60 °C with the heating rate of 5 °C per minute.

Thermogravimetric analysis of selected copolymers was performed on TA instrument, TGA Q50 (New Castle, DE, USA) using a flow of nitrogen stream. The measurements were conducted within the temperature range of 25–600 °C, where heating rate of 10 °C per minute was used.

Waste cooking oil was used as raw material for the production of biopolymers. Triacylglycerides (TAG’s) were separated from waste cooking oil by silica gel column chromatography using 3% ethyl acetate in n-hexane and used for trans-esterification.

### 2.3. Transesterification of TAG’s

TAG’s (60 g, 0.068 moles) were placed in a 250 mL round bottom flask and dissolved in 40 mL of methanol. KOH (40 mg) solution in methanol (5 mL) was added to the reaction mixture and stirred vigorously at 45 °C for two hours. Progress of the reaction was monitored by TLC using ethyl acetate and n-hexane (1:9) as solvent. After completion of reaction, 150 mL of DCM was added to the reaction mixture and washed with water to remove KOH and methanol. Solvent was removed over rotary evaporator and dried over anhydrous sodium sulphate. 

### 2.4. Epoxidation of Methyl Esters

In a 100 mL round bottom flask fitted with stirrer, methyl ester of TAG’s (1 g) was introduced and dissolved in 15 mL of dichloromethane at 0 °C. 3-chloroperoxybenzoic acid was added gradually as the reaction was exothermic. This reaction mixture was stirred at room temperature and the progress of reaction was monitored by TLC. The reaction was completed within half an hour. The excess peroxybenzoic acid from the reaction mixture was filtered out and crude product was purified over column chromatography using 4% ethyl acetate in n-hexane as an eluent, where colourless epoxy methyl ester was obtained with 47% yield.

### 2.5. Synthesis of Polymers

A mixture of phthalic anhydride (142 mg, 0.961 mol) and (salen)Cr^III^Cl catalyst (0.0096–0.00096 mol) was weighed in reaction vial fitted with magnetic stirrer under nitrogen atmosphere. Co-catalyst n-Bu_4_NCl/DMAP (0.0076–0.00096 mol) and epoxide (300 mg, 0.961 mol) were added and again purged with nitrogen. The reaction was carried out in a microwave oven (set power: 200 W) for 30 min at 100 °C. A viscous polymer formed in the reaction vial. The reaction was quenched by exposing the reaction contents to air and by dissolving in THF. This solution was precipitated in cold methanol to get purified copolymer.

## 3. Results and Discussion

**Scheme 1:**
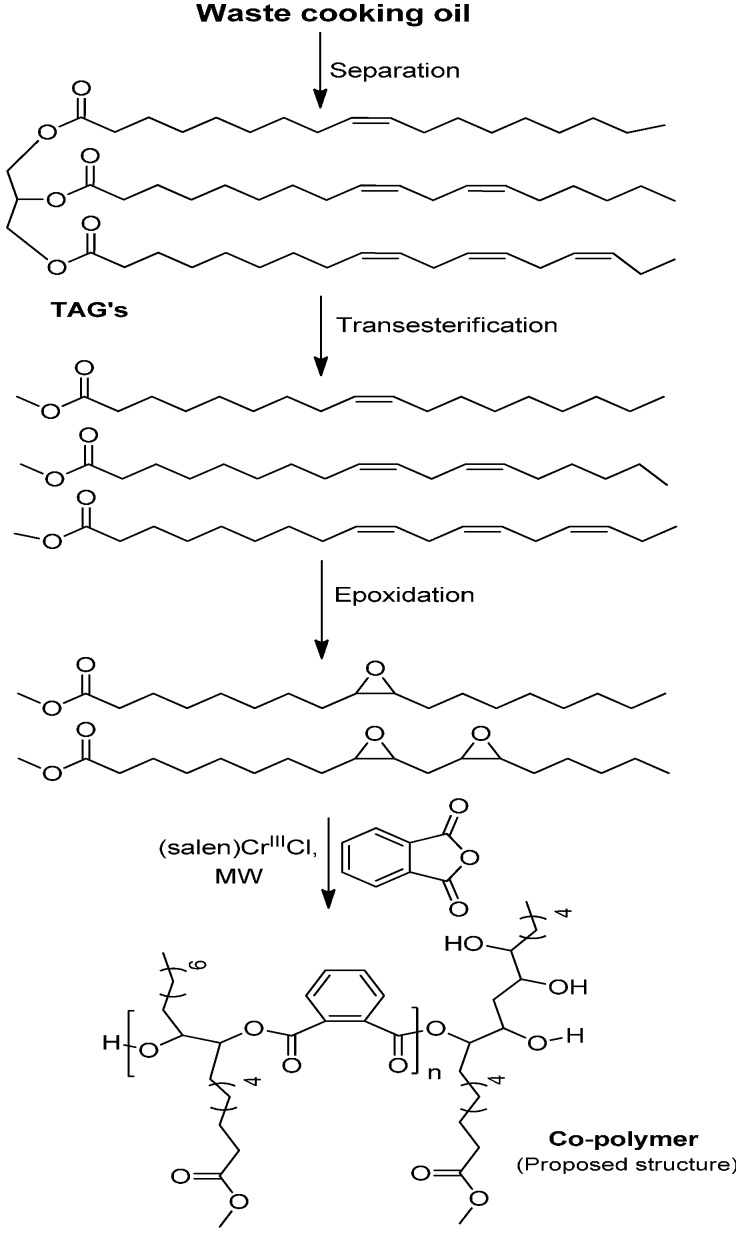
Copolymerization of epoxidized fatty esters derived from waste cooking with phthalic anhydride using catalyst (Salen)Cr^III^Cl and co-catalysts n-Bu_4_NCl and DMAP.

The ring opening copolymerization of epoxy fatty esters derived from waste cooking oil and phthalic anhydride was carried out in the presence of (salen)Cr^III^Cl as catalyst, while two different co-catalysts n-Bu_4_NCl and DMAP were used individually under microwave irradiation. Biermann et al. already reported the copolymerization of methyl 9,10-epoxystearate with phthalic anhydride by conventional heating at higher temperature of 110–116 °C with longer reaction times [14]. Microwave technology is a green method for chemical synthesis because of its high efficiency and homogeneous heating. The microwaves lead to enhanced reaction rates, yields, and fewer by-products with dramatic shortening of reaction times [32]. Using microwave irradiation, firstly we undertook the copolymerization of epoxide and anhydride without any solvent using (salen)Cr^III^Cl as a catalyst and n-Bu_4_NCl as co-catalyst by varying temperature, time, catalyst and co-catalyst ratio (Scheme 1, Table 1). Initially, the reactions were performed with catalyst and co-catalyst ratio of 1/0.8 at 100 °C for different time intervals ranging from 5 min to 30 min, where 1:1 epoxy and anhydride ratio were used (Table 1, entry 1–4). The results given in Table 1 illustrate that reactions with increased reaction times (30 min) provide copolymer with higher molecular weight (*M*w = 6000 g/mol) than the reaction with shorter time (5 min) giving *M*w = 3900 g/mol, while slightly higher dispersity (D = 1.39–1.92) values for all copolymers were obtained. By keeping the same catalyst and co-catalyst concentration, when reactions were performed with different ratios (2:1, 1:2 and 7:3) of epoxide and anhydride respectively, no increase in molecular weight of copolymers was seen (Table 1, entry 5–7). It has been noticed that increase in a ratio of epoxy fatty ester/anhydride results in a lower molecular weight, which could be attributed to longer hydrophobic chain of epoxy fatty ester causing more hindrance during polymerization. After that, the reactions were further evaluated at 100 °C by lowering the catalyst and co-catalyst ratio from 1/0.8 to 0.1/0.1 to get their optimum concentration (Table 1, entry 8–12). It was found that lowering the catalyst and co-catalyst ratio results in a gradual decrease of copolymers average molecular weights from 4950 g/mol to 3100 g/mol (Table 1, entry 8–12). No improvement in molecular weight was observed even when reaction time was increased from 30 to 50 min (Table 1, entry 10 and 11). From these results, the catalyst and co-catalyst ratio of 0.5/0.8 was found to be optimal for further polymerization reaction. Moreover, using an optimized concentration of catalyst and co-catalyst, the reactions were investigated at different temperatures (70, 80 and 90 °C) for a time span of thirty minutes. So, lowering the reaction temperature to 90 °C and 80 °C results in an improved *M*w of 6750 g/mol and 6050 g/mol with narrow dispersity values (D = 1.25 and 1.5) respectively (Table 1, entry 13 and 14). However, no polymer formation was observed at 70 °C. These results suggest that 90 °C is a suitable temperature for copolymerization reactions. So after optimizing the ratio of catalyst/co-catalyst, epoxide/anhydride and temperature, the reaction was further performed for a longer reaction time (50 min), but no increase in molecular weight was observed (Table 1, entry 16). This shows that increasing the reaction times more than 30 min does not have any significant effect on the average molecular weight of copolymers.

Furthermore, 4-dimethylaminopyridine (DMAP) as a co-catalyst instead of Bu_4_NCl was investigated for ring opening copolymerization reaction at 100 °C for 30 min using equimolar ratio of epoxy ester and phthalic anhydride. Initially the reaction was performed with 1/0.8 equivalence of (salen)Cr^III^Cl and DMAP, a copolymer with good molecular weight (*M*w = 6050) and narrow dispersity value (D = 1.12) was obtained (Table 2, entry 1). Further reactions were performed by gradually decreasing the catalyst and co-catalyst ratio from 1/0.8 to 0.1/0.1 at 100 °C (Table 2, entry 2–6). Co-polymer formation was observed in all cases, even when the catalyst and co-catalyst ratio was lowered to 0.1/0.1 equivalence. The obtained molecular weights for most of the copolymer was moderately higher (above 6600 g/mol) with narrower dispersity value (less than 1.17) as compared to those reactions where Bu_4_NCl was used as a co-catalyst (Table 1). These results suggest that the catalyst and co-catalyst ratio of 0.2/0.4 is the optimal concentration as it provides the copolymer with highest molecular weight *M*w = 6950 g/mol and low dispersity value D = 1.08 (Table 2, entry 5). By using this optimized concentration of catalyst and co-catalyst, further reactions were carried out at 90 °C and 80 °C. At 90 °C, the obtained copolymer has molecular weight of 6800 g/mol and dispersity value of 1.07 (Table 2, entry 7), while at 80 °C, the formation of copolymer also occurred with molecular weight of 5500 g/mol and dispersity value of 1.08 (Table 2, entry 8). These results illustrate that the copolymer obtained at 80 °C has lower molecular weight as compared to those prepared at 90 and 100 °C. These results indicate that the ring opening copolymerization reactions below 80 °C will result in a low molecular weight polymer. From all these results (Table 1 and Table 2), it can be concluded that co-catalyst DMAP is more active with (salen)Cr^III^Cl catalyst even when used in low concentration, as it provides a copolymer with higher molecular weights and narrow dispersity values as compared to co-catalyst Bu_4_NCl.

**Figure 1:**
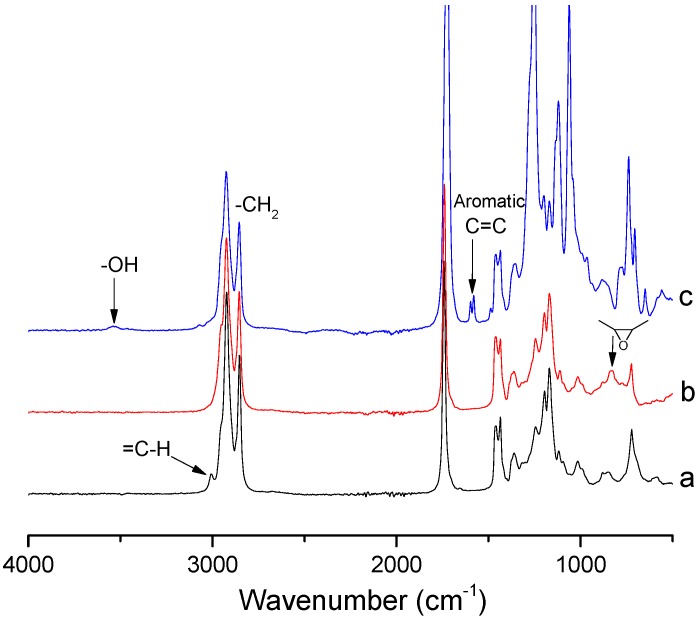
Attenuated total reflection-Fourier transform infrared spectroscopy (ATR-FTIR) spectra of (**a**) fatty esters; (**b**) epoxidized fatty esters and (**c**) polyesters.

The synthesized polyesters were characterized and evaluated by different analytical techniques such as ATR-FTIR, ^1^H-NMR, GPC, TGA and DSC analysis. The ATR-FTIR spectra of fatty esters derived from waste cooking oil (a), expoxidized fatty esters (b) and synthesized polyester (c) are given in Figure 1. The absorption band at 3005 cm^−1^ in the spectrum of fatty esters (a) is attributed to unsaturated alkenes (–C=C–H), which has been completely disappeared in the spectrum of epoxidized fatty ester (b). This indicates the complete consumption of unsaturated alkenes into epoxy groups. The appearance of a new band at 831 cm^-1^ in the spectrum of epoxidized fatty ester (b) corresponds to epoxy groups [33], which is another indication of the conversion of unsaturated alkenes into epoxy groups. During ring opening copolymerization of epoxy fatty esters with phthalic anhydride, the epoxy ring opens up and results in the formation of a hydroxyl functional group. In the polyester spectrum (c), an absorption band at 3531 cm^−1^ corresponding to the hydroxyl group has appeared, while the peak at 831 cm^−1^ due to epoxy groups has completely disappeared. Another new band at 1586 cm^−1^ that belongs to the stretching vibration of aromatic carbon-carbon double bond (–C=C–) was also observed. The presence of both these new peaks and absence of a peak affiliated to epoxy groups in the polyester spectrum (c) confirms the formation of polyesters and addition of phthalic anhydride during ring opening polymerization reactions.

**Figure 2:**
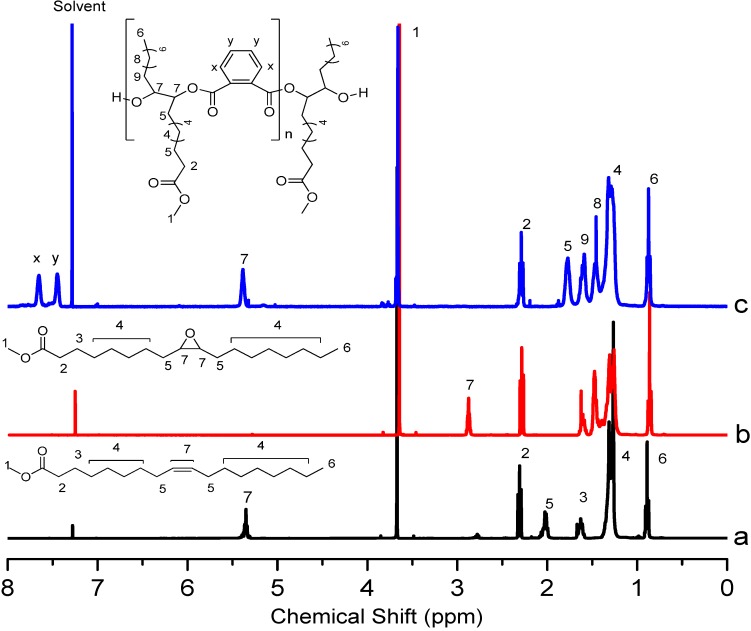
^1^H NMR spectra of (**a**) fatty esters; (**b**) epoxidized fatty esters and (**c**) polyesters.

Epoxidation of fatty esters derived from waste cooking oil and its polymerization with phthalic anhydride was further evaluated with ^1^H-NMR spectroscopy as shown in Figure 2. The ^1^H NMR signal at 5.36 ppm due to unsaturated protons of fatty esters (a) has completely disappeared or shifted towards the upfield region (δ = 2.85 ppm, labelled as “*7*” attributed to epoxy protons) in the spectrum of epoxy fatty esters (b). This represents the successful epoxidation of all unsaturated double bonds into epoxy groups. In the polyester spectrum (c), the signals associated to epoxy ring protons have completely disappeared, indicating that all the epoxy rings have been opened up and consumed during polymerization. The presence of two new signals in the aromatic region at 7.65 ppm and 7.43 ppm labelled as “*x*” and “*y*” corresponds to aromatic protons of phthalic anhydride confirms its copolymerization with the epoxy ring of fatty esters (Figure 2c).

**Figure 3:**
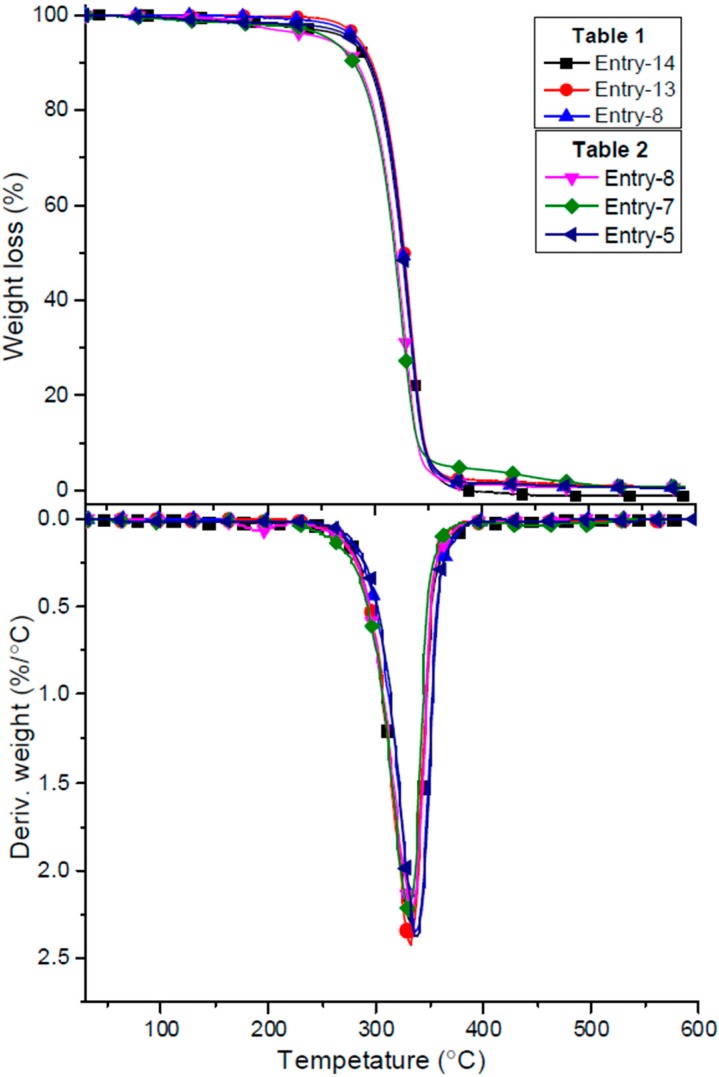
TG and DTG plots of polyesters (Table 1, entry 8, 13, and 14) obtained by using Bu_4_NCl and polyesters (Table 2, entry 5, 7, and 8) obtained by using DMAP as a co-catalyst.

DSC thermograms of selective copolymers have also been run (Figure S1) and detail has been discussed in electronic supplementary information (ESI). TG (thermogravimetric) and DTG (derivative thermogravimetric) plots of some selected polyesters were studied to investigate their thermal stability and degradation behavior (Figure 3). It is obvious from Figure 3, that major weight loss (%) of all polyesters is in the temperature range of 250–370 °C. The polyesters labelled as entry 7 and 8 (Table 2) have shown their initial weight loss starting from 230 °C, while all other polyesters displayed at 270 °C, which could be attributed to the difference in their molecular weights. The final weight loss of polyesters entry 7 and 8 (Table 2) was found to be 2%–4% less than all other polyesters. These variations could be assigned to their different molecular weight. DTG curves of polyesters illustrate the temperature where maximum weight loss (T_max_) for polyesters was observed. The polyesters labeled as entry 8 (Table 1) and entry 5 (Table 2) displayed maximum weight loss at 338 °C as compared to all other polyesters (T_max_ = 332 °C). Both of the polyesters entry 8 (Table 1) and entry 5 (Table 2) exhibited approximately 6 °C higher stability than the other polyesters. The higher stability of polyester (Table 2, entry 5) could be attributed to its higher molecular weight (*M*w = 6950 g/mol) and narrow dispersity value (D = 1.08) as compared to all other polyesters synthesized by using DMAP as a co-catalyst (Table 2). While an anomalous behavior was observed by the polyester labeled as entry 8 (Table 1), as even having low molecular weight (*M*w = 4950 g/mol) and moderate dispersity value (D = 1.54) exhibit slightly higher thermal stability (6 °C) than the other polyesters prepared by using Bu_4_NCl as a co-catalyst having higher molecular weight and lower dispersity values (Table 1).

## 4. Conclusions

In conclusion, polyesters were successfully prepared from fatty esters derived from waste cooking oil and phthalic anhydride using (salen)Cr^III^Cl as catalyst and n-Bu_4_NCl/DMAP as a co-catalyst under microwave irradiation. The polyester with highest molecular weight (*M*w = 6750) and narrow dispersity value (D = 1.25) was obtained when (salen)Cr^III^Cl/n-Bu_4_NCl was used with 0.5/0.8 equivalence at 90 °C for 30 min. Under similar conditions at 100 °C, when DMAP was used as a co-catalyst, a polyester with improved molecular weight (*M*w = 6950) and narrow dispersity value (D = 1.08) was obtained even at reduced concentrations (0.2/0.4) of (salen)Cr^III^Cl/DMAP respectively. So DMAP was found to be the most suitable co-catalyst with (salen)Cr^III^Cl to be used for ring opening co-polymerization. The polyesters were synthesized via ring opening copolymerization under microwave conditions at lower temperature leading to reduced polymerization times (hours to minutes) compared with classical thermal polymerization. In conclusion, a renewable resource (waste/recycled cooking oil) was used to prepare biopolymers with good molecular weight having great potential to replace petroleum based products in the future. This study will contribute greatly to making waste cooking oil useful for the polymer industry.

## Figures and Tables

**Table 1:** Co-polymerization of epoxide and phthalic anhydride using (salen)Cr^III^Cl as catalyst and n-Bu_4_NCl as co-catalyst.

Entry	Catalyst/Co-Catalyst Ratio	Temperature (°C)	Time (min)	Epoxy/Anhydride	*M*w (g/mol)	D
1	1/0.8	100	30	1:1	6000	1.83
2	1/0.8	100	20	1:1	4950	1.42
3	1/0.8	100	10	1:1	4700	1.39
4	1/0.8	100	5	1:1	3900	1.92
5	1/0.8	100	30	2:1	4500	1.39
6	1/0.8	90	40	1:2	5300	1.18
7	1/0.8	100	30	7:3	3350	1.45
8	0.5/0.8	100	30	1:1	4950	1.54
9	0.4/0.6	100	30	1:1	4800	1.43
10	0.3/0.5	100	50	1:1	4000	1.28
11	0.2/0.4	100	50	1:1	3900	1.28
12	0.1/0.1	100	30	1:1	3100	1.44
13	0.5/0.8	90	30	1:1	6750	1.25
14	0.5/0.8	80	30	1:1	6050	1.5
15	0.5/0.8	70	30	1:1	0	-
16	0.5/0.8	90	50	1:1	5400	1.15

**Table 2:** Co-polymerization of epoxide and phthalic anhydride using (salen)Cr^III^Cl as catalyst and DMAP as co-catalyst.

Entry	Catalyst/Co-Catalyst Ratio	Temperature (°C)	Time (min)	Epoxy/Anhydride	*M*w (g/mol)	D
1	1/0.8	100	30	1:1	6050	1.12
2	0.5/0.8	100	30	1:1	6400	1.28
3	0.4/0.6	100	30	1:1	6600	1.25
4	0.3/0.5	100	30	1:1	6900	1.08
5	0.2/0.4	100	30	1:1	6950	1.08
6	0.1/0.1	100	30	1:1	6750	1.17
7	0.2/0.4	90	30	1:1	6800	1.07
8	0.2/0.4	80	30	1:1	5500	1.08

## Supplementary Materials

The following are available online at www.mdpi.com/1996-1944/10/3/315/s1, Figure S1: DSC thermograms of selected polyesters Table 1, entry 1, and Table 2, entry 2, & 3.
