# Supplementary material for: Microwave-Assisted Catalytic Synthesis of Bio-Based Copolymers from Waste Cooking Oil
Source: Materials (Basel). 2017 Mar 20;10(3):315. doi: 10.3390/ma10030315 (PMC5503379; doi:10.3390/ma10030315)
Supplement: Supplementary file 1 [file materials-10-00315-s001.pdf]

Electronic Supporting Information (ESI)

Microwave-assisted catalytic synthesis of bio-based copolymers from waste cooking oil

Mahrzadi Noureen Shahi [1,2], Muhammad Arshad [1] and Aman Ullah [1,*]

[1] Department of Agricultural, Food and Nutritional Sciences, University of Alberta, Edmonton, AB T6G 2P5, Canada; mahrzadi@ualberta.ca (M.N.S.); arshad4@ualberta.ca (M.A.)
[2] Department of Chemistry, The Islamia University of Bahawalpur, Bahawalpur 63100, Pakistan
* Correspondence: ullah2@ualberta.ca; Tel.: +1-780-492-4845

Differential Scanning Calorimetry (DSC):

Figures S1 shows the DSC thermo grams of 2nd heating run of the selected polymers obtained by MW assisted copolymerization. It is evident from thermograms that these biopolymers generally show transitions close to -20°C which could be the glass transitions of these biopolymers. The polymer synthesized using DMAP as co-catalyst entry 3 table 2 clearly displayed two additional peaks, one endothermic peak which could be melting and another exothermic which could be due to crystallization.

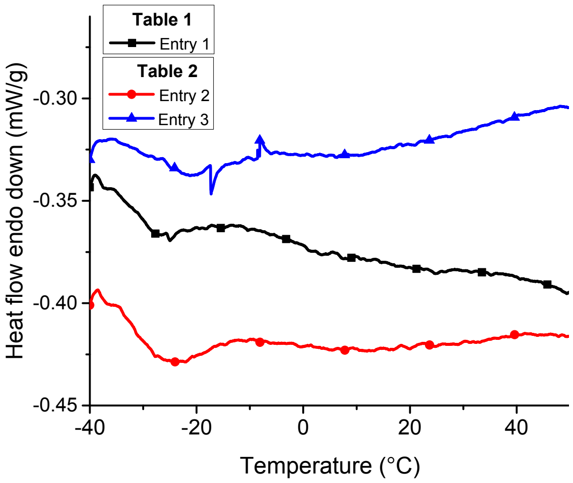

Figure S1. DSC thermograms of selected polyesters Table 1, entry 1, and Table 2, entry 2, & 3.
